# Comparative Diagnostic Assessment of Karyotyping, Microarray, and Whole Exome Sequencing in Genetically Associated Fetal Growth Restriction

**DOI:** 10.3390/diagnostics16020312

**Published:** 2026-01-18

**Authors:** Libing Luo, Chunchun Chen, Cindy Ka Yee Cheung, Yanyan Li, Xiaoying Dai, Ting Zeng, Ying Wang

**Affiliations:** 1 Prenatal Diagnosis Centre, The University of Hong Kong—Shenzhen Hospital, Shenzhen 518053, China; chencc@hku-szh.org (C.C.); cindycky@hku-szh.org (C.K.Y.C.); liyy@hku-szh.org (Y.L.); daixy3@hku-szh.org (X.D.); zengt8@hku-szh.org (T.Z.); 2 Shenzhen Clinical Research Center for Rare Diseases, Shenzhen 518053, China; 3 Department of Obstetrics and Gynaecology, LKS Faculty of Medicine, The University of Hong Kong, Hong Kong, China; 4 Department of Obstetrics and Gynaecology, The University of Hong Kong—Shenzhen Hospital, Shenzhen 518053, China; wangy10@hku-szh.org

**Keywords:** fetal growth restriction (FGR), karyotype analysis, chromosomal microarray analysis (CMA), whole exome sequencing (WES), copy number variations (CNVs), uniparental disomy (UPD)

## Abstract

**Background:** Fetal growth restriction (FGR) is a significant obstetric complication associated with increased perinatal morbidity and long-term developmental risks. Despite advances in prenatal diagnosis, the genetic etiology of isolated FGR remains incompletely characterized, complicating genetic counseling and clinical management. **Objective:** This study aimed to systematically evaluate the genetic causes of isolated FGR by integrating karyotyping, chromosomal microarray analysis (CMA), and trio-based whole exome sequencing (trio-WES) and to assess the incremental diagnostic yield of this sequential approach. **Methods:** A retrospective cohort of 153 fetuses with isolated FGR (diagnosed by ultrasound between February 2018 and July 2024) underwent karyotyping and CMA. Cases with normal results from both tests (*n* = 50) were subsequently analyzed by trio-WES. **Results:** Karyotyping identified chromosomal abnormalities in three cases (2.0%). CMA detected pathogenic/likely pathogenic copy number variations (CNVs) or uniparental disomy (UPD) in twelve cases (7.8%), including the three karyotypic abnormalities and nine additional cases (5.9% incremental yield). Trio-WES performed on 50 CMA-negative cases identified pathogenic or likely pathogenic variants in 12 cases (24%). Among these, seven cases (14% of the WES subgroup) harbored variants directly causative of FGR, including one case of UPD(6) missed by CMA alone. Additionally, trio-WES revealed seven incidental pathogenic/likely pathogenic variants not directly linked to FGR and identified one case in which FGR was attributed to maternal hyperphenylalaninemia. **Conclusions:** The sequential application of CMA and trio-WES significantly improves the diagnostic yield for isolated FGR. Trio-WES proved particularly valuable in detecting UPD and single-gene variants missed by CMA alone and in revealing contributory maternal genetic conditions. These findings support the integration of advanced genetic testing into the diagnostic workup for isolated FGR to enhance etiological diagnosis, facilitate comprehensive genetic counseling, and inform multidisciplinary management.

## 1. Introduction

Fetal growth restriction (FGR) is a common obstetric complication characterized by fetal growth below its genetic growth potential, typically indicated by an estimated fetal weight (EFW) or abdominal circumference below the 10th percentile for the corresponding gestational age [1,2,3]. FGR significantly increases the risk of perinatal morbidity and mortality, leading to short-term issues such as fetal distress and stillbirth, as well as long-term consequences, including developmental delay and metabolic syndrome in adulthood [4,5,6,7,8]. The etiological mechanisms of FGR are complex, involving multiple factors related to the mother, placenta, umbilical cord, and fetus [5,9,10,11]. Genetic abnormalities play a crucial role in the occurrence and prognosis of FGR, with approximately 15–20% of cases associated with chromosomal anomalies, including aneuploidies, copy number variations (CNVs), and epigenetic alterations [11,12,13,14,15]. Common etiologies include aneuploidies such as trisomy 21, trisomy 13, and trisomy 18, with FGR observed in approximately 90% of affected fetuses [16].

Advances in genetic diagnostic techniques have enhanced the detection rate of chromosomal abnormalities in FGR. Conventional karyotyping can detect chromosomal aneuploidies and large-scale structural variations, but it has limited resolution for small imbalances. Array comparative genomic hybridization (aCGH) and single-nucleotide polymorphism (SNP) microarrays have improved the detection capability for chromosomal abnormalities; SNP microarrays can also identify CNVs and uniparental disomy (UPD). Compared with conventional karyotyping, these technologies significantly increase the detection rate [16,17,18,19,20,21,22,23]. The American College of Obstetricians and Gynecologists (ACOG) and the Society of Maternal-Fetal Medicine (SMFM) recommend chromosomal microarray analysis (CMA) as the primary prenatal diagnostic tool for fetal anomalies [20,24]. However, despite progress in detecting chromosomal abnormalities, the diagnostic yield of CMA for isolated FGR ranges from 0% to 26.3% (mean 6.4%), highlighting the need for further genetic investigation [9,15,25,26,27]. Whole-exome sequencing (WES) has become an important tool for diagnosing monogenic diseases by identifying single-nucleotide variants (SNVs) and insertions/deletions (indels), potentially increasing diagnostic yield by 10–31% in fetuses with structural anomalies but negative CMA results [28,29]. Recent evidence indicates that WES can detect genetic abnormalities in from 10% to 15% of isolated FGR cases [30,31]. However, studies on the genetic etiology of isolated FGR without accompanying structural anomalies remain limited. This knowledge gap complicates clinical genetic counseling and pregnancy decision-making. This cohort study specifically excludes fetuses with ultrasonographically detectable structural anomalies, thereby focusing on the unique subgroup with only growth restriction identified prenatally, in contrast to cases in which FGR is accompanied by or precedes the development of structural abnormalities.

This study aims to investigate the genetic etiology of isolated FGR through the combined application of G-banded karyotype analysis, CMA, and trio-WES. The findings will be utilized to evaluate the clinical utility of the aforementioned genetic tests, optimize diagnostic pathways, and provide more targeted guidance for clinical management.

## 2. Materials and Methods

### 2.1. Patient Enrollment

**Figure 1:**
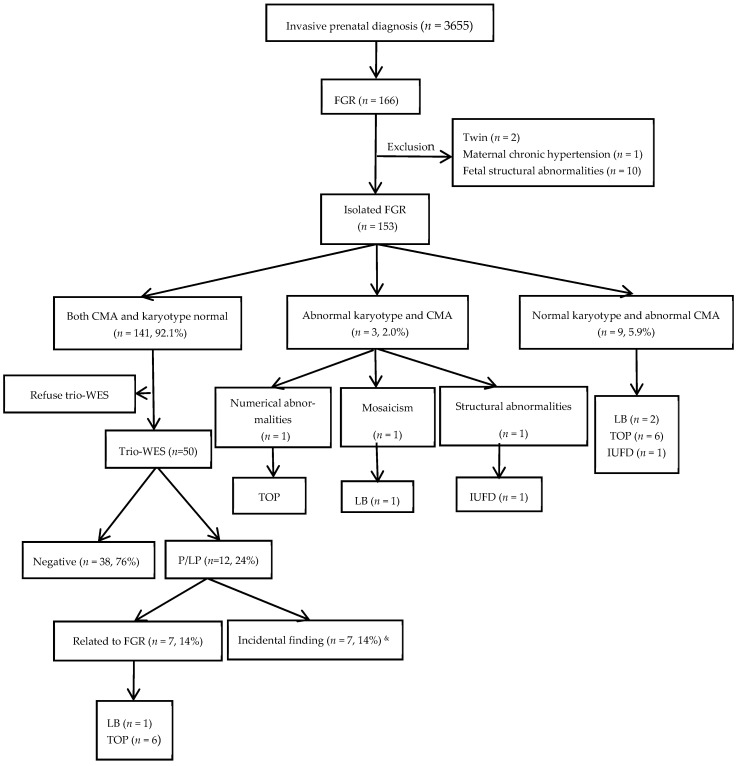
Flowchart of the cohort in isolated fetal growth restriction (FGR) fetuses. CMA, chromosomal microarray analysis; P/LP, pathogenic/likely pathogenic; TOP, termination of pregnancy; Trio-WES, trio-based whole-exome sequencing; LB, live-born; IUFD, intrauterine fetal demise. &: Among the seven incidental findings, two coexisted with variants causally associated with FGR in the same cases: in Case 16, a pathogenic *IHH* variant linked to FGR was accompanied by an incidental *ENPP1* variant, while in Case 20, an *FGFR2* incidental finding occurred alongside UPD6, which was identified as the genetic cause of FGR.

A retrospective analysis was conducted on 153 fetuses diagnosed with isolated FGR by ultrasound at the Prenatal Diagnosis Center of the University of Hong Kong–Shenzhen Hospital between February 2018 and July 2024, all of which subsequently underwent invasive prenatal diagnosis (Figure 1). During this period, a total of 3655 invasive prenatal diagnostic procedures were performed at the center, among which 166 cases were diagnosed with FGR. Ultimately, 153 cases of isolated FGR were included in the study. All cases met the following criteria: (1) Diagnostic criteria for FGR: According to the standards for Asian populations set by the National Institute of Child Health and Human Development (NICHD), after correcting gestational age with early pregnancy ultrasound, with ultrasound measurements of EFW or abdominal circumference were less than the 10th percentile for fetuses of the same gestational age. (2) No fetal structural abnormalities were detected on ultrasound. (3) No severe maternal medical conditions, such as chronic hypertension or insulin-dependent diabetes mellitus, were detected. (4) Singleton pregnancy occurred. All participants signed informed consent, and this study was approved by the Ethics Committee of the University of Hong Kong–Shenzhen Hospital (hkuszh2025258).

After genetic counseling, all cases underwent amniocentesis, with concurrent chromosomal karyotype analysis and chromosomal microarray testing. Among the 141 couples with negative karyotype and microarray results, 50 couples opted for further trio-WES following genetic counseling. Peripheral blood samples from both parents were collected for joint analysis.

### 2.2. Amniotic Fluid Sampling and DNA Extraction

Under ultrasound guidance, amniocentesis was performed to collect 40 mL of amniotic fluid. Of this, 10 mL was allocated for CMA, methylation-specific multiplex ligation-dependent probe amplification (MS-MLPA), and maternal cell contamination testing; 20 mL was equally divided into two independent culture systems for standard G-banding karyotype analysis; the remaining 10 mL was retained for potential further genetic testing (e.g., trio-WES).

Peripheral blood (PB) and amniocyte DNA were extracted using the QIAamp DSP DNA Blood Mini Kit and the Puregene cell kit (QIAGEN, Hilden, Germany), respectively, from 200 µL of PB and 10 mL of amniotic fluid, according to the manufacturer’s protocol (www.qiagen.com), and 50 µL of DNA was eluted in TE buffer.

Maternal cell contamination was investigated using polymerase chain reaction (PCR)-based short tandem repeat (STR) analysis. Cultured amniocytes were used for downstream analysis if maternal contamination was detected.

### 2.3. CMA and Trio-WES

CMA was sent out to Shenzhen Maternity and Child Healthcare Hospital before 2019 using the CytoScan 750K chip kit (Affymetrix, Santa Clara, CA, USA) containing CNV and SNP probes. From 2019 onward, CMA was performed at the HKU-SZH prenatal diagnostic laboratory using the SurePrint G3 Human CGH Microarray 8x60K (Agilent, Santa Clara, CA, USA) with/without ME034 MS-MLPA (MRC Holland, Amsterdam, The Netherlands). Data analysis was performed using the CHAS software version 4.2.80 (Affymetrix, USA) or the CytoGenomics Software v5.4 (Agilent, USA) in conjunction with relevant databases, including DGV, Decipher, UCSC, and ClinGen databases.

Samples for WES testing were sent to the commercial laboratory BGI. The DNA library was sequenced on the MGISEQ-2000 platform (MGI Tech Co., Ltd., Shenzeng, China) with a targeted sequencing depth of >300×; the sequencing depth of >20× was 98%. The analysis was conducted using an internal bioinformatics pipeline, which included aligning reads to the GRCh37/hg19 genome assembly, variant calling, annotation, and comprehensive variant filtering. The annotation databases primarily included gnomAD (http://gnomad.broadinstitute.org/), the 1000 Genomes Project (http://browser.1000genomes.org), BGI’s database, dbSNP (http://www.ncbi.nlm.nih.gov/snp), SIFT (http://sift.jcvi.org), MutationAssessor (http://mutationassessor.org/r3/), HPO (https://hpo.jax.org/app), OMIM, ClinVar databases, etc. Variants were classified into five categories according to the ACMG guidelines [32] for the interpretation of genetic variants: “pathogenic (P),” “likely pathogenic (LP),” “benign (B),” “likely benign (LB),” and “variant of uncertain significance (VUS).”

## 3. Results

### 3.1. Chromosomal Karyotype and Chromosomal Microarray Analysis Results

A total of three abnormal cases were detected by karyotype analysis (Table 1), yielding a detection rate of 2.0% (3/153). Among these, one case was a Turner syndrome mosaicism (6%), one case was trisomy 21, and one case had a partial deletion of chromosome 11.

CMA identified 12 pathogenic or likely pathogenic copy number variants (CNVs) and uniparental disomy (UPD) (Table 2), with a detection rate of 7.8% (12/153). Among these, three cases exhibited abnormal karyotypes, and the CMA results were consistent with the karyotype analysis findings (Table 1). Among the 150 fetuses with normal karyotypes, CMA detected nine cases with chromosomal abnormalities, including six cases of de novo chromosomal microdeletions (Cases 5, 6, 8, 10, 11, and 12), one case of a maternally inherited 17p12 microduplication (Case 7), and two cases of UPD (Cases 4 and 9), resulting in a detection rate of 6.0% (9/150). These chromosomal abnormalities involved 1q43-q44 microdeletion syndrome (Case 6), Xp22.33p22.31 microdeletion associated with Leri–Weill dyschondrosteosis (Case 8), 15q13.3 microdeletion syndrome (Case 10), 19q13.11q13.12 microdeletion (Case 11), UPD(6) (Case 9), segmental UPD(16) (Case 4), 14q12 microdeletion (Case 5), 17p12 microduplication associated with Charcot–Marie–Tooth disease type 1A (CMT1A) (Case 7), and 15q11.2 microdeletion syndrome (Case 12).

### 3.2. Results of Trio-WES

Among 50 cases with negative karyotype and CMA results, 12 cases (12/50, 24%) were found to harbor pathogenic or likely pathogenic variants (Table 3). A total of ten pathogenic and four likely pathogenic variants were identified, involving 13 genes; an additional case (Case 20) exhibited UPD(6) that was not detected by CMA. Of these, seven cases (7/50, 14%) carried variants directly associated with FGR, including five cases of autosomal dominant disorders (Cases 13, 14, 16, 17, and 18) with mutations in the *WHSC1*, *FGFR3*, *IHH*, *TUBB2B*, and *BRPF1* genes, respectively; one case of an autosomal recessive disorder (Case 15) caused by compound heterozygous mutations in the *LIG4* gene; and one case of UPD(6) (Case 20). Furthermore, trio-WES identified seven pathogenic or likely pathogenic variants not directly linked to the FGR phenotype, involving the *SMAD6*, *FLNB*, *MSH2*, *BRIP1*, *ENPP1*, *FGFR2*, and *PAH* genes. Notably, two cases were found to carry both incidental variants and pathogenic variants associated with FGR. Case 16 harbored heterozygous mutations *IHH*:c.84C>A (p.Cys28*) and *ENPP1*:c.783C>G (p.Tyr261*). The fetal phenotype included a short femur, normal head circumference and abdominal circumference, and an estimated fetal weight below the 10th percentile for gestational age, consistent with a diagnosis of FGR. The paternally inherited heterozygous mutation *IHH*:c.84C>A (p.Cys28*) was classified as a likely pathogenic variant. The father, with a height of 170 cm, exhibited clinical features of brachydactyly, aligning with the phenotype of brachydactyly type A1 (OMIM 112,500) associated with IHH, suggesting a correlation between this heterozygous mutation and the observed short long bones in the fetus. The concurrently present heterozygous mutation *ENPP1*:c.783C>G (p.Tyr261*) was maternally inherited. The mother presented with keratosis pilaris on her legs, consistent with the clinical manifestations of Cole disease associated with the *ENPP1* gene. This variant was unrelated to FGR and was considered an incidental finding. Case 20 involved a maternal uniparental disomy of chromosome 6—a confirmed genetic etiology for FGR. Simultaneously, an *FGFR2* variant (c.1032G>A, p.Ala344=) associated with craniosynostosis syndrome but unrelated to growth restriction was detected. Furthermore, a heterozygous *PAH*:c.1197A>T variant was detected in Case 19. This variant is pathogenic and was inherited from the mother. Trio-WES revealed that the mother carries compound heterozygous pathogenic variants, *PAH*:c.1197A>T and c.1238G>C. The mother’s blood phenylalanine level was measured at 757.42 µmol/L (reference range: 20–120 µmol/L), leading to a diagnosis of hyperphenylalaninemia in the mother herself. The mother had a history of one early miscarriage, one pregnancy termination due to fetal cardiac defects, and one full-term delivery (the offspring had intellectual disability). Genetic testing of family members had not been performed prior to this prenatal diagnosis. Although the heterozygous *PAH*:c.1197A>T variant in this fetus was not the cause of the FGR (it was an incidental finding), trio-WES analysis indicated that the etiology of the FGR in this pregnancy was maternal hyperphenylalaninemia. The mother’s previous three adverse pregnancy outcomes are highly likely associated with her undiagnosed and uncontrolled hyperphenylalaninemia, which is consistent with the characteristics of adverse pregnancy outcomes reported in the literature for pregnant women with uncontrolled hyperphenylalaninemia [33,34,35,36,37,38].

### 3.3. Pregnancy Outcome

Among the three cases with abnormal karyotypes, Case 1 was a mosaic Turner syndrome, delivered at 38 weeks and 4 days of gestation with a neonatal birth weight of 2690 g. Case 2 was a trisomy 21 syndrome, and the pregnancy was terminated. Case 3 had a partial deletion of chromosome 11, resulting in intrauterine fetal demise. Among the additional nine cases of chromosomal abnormalities detected by CMA, five opted for pregnancy termination. These cases included: 1q43-q44 microdeletion syndrome (Case 6), Xp22.33p22.31 microdeletion associated with Leri–Weill dyschondrosteosis (Case 8), 15q13.3 microdeletion syndrome (Case 10), 19q13.11q13.12 microdeletion (Case 11), and UPD(6) (Case 9). Case 5, involving a 14q12 microdeletion, resulted in subsequent intrauterine fetal demise. Three pregnancies were continued: Case 7, carrying a maternally inherited 17p12 microduplication associated with Charcot–Marie–Tooth disease, was delivered preterm at 34 weeks due to preeclampsia, with a birth weight of 1830 g and hypospadias; Case 12, diagnosed with 15q11.2 microdeletion syndrome, was delivered at 39 weeks and 3 days of gestation with a birth weight of 2540 g; and Case 4, with segmental UPD(16), was delivered at 37 weeks and 5 days of gestation with a birth weight of 1660 g. All three live-born infants demonstrated normal development during the follow-up period of 1 to 3 years.

Of the seven WES-identified cases with pathogenic or likely pathogenic variants related to the FGR phenotype, six opted to terminate the pregnancy. These included cases with autosomal dominant genetic diseases caused by mutations in *WHSC1*, *FGFR3*, *TUBB2B*, and *BRPF1* (cases 13, 14, 17, and 18), an autosomal recessive genetic disease caused by compound heterozygous mutations in the *LIG4* gene (Case 15), and a case of maternal UPD(6) combined with a pathogenic mutation in the *FGFR2* gene (Case 20). Case 16, with a heterozygous *IHH*:c.84C>A(p.Cys28*) and a heterozygous *ENPP1*:c.783C>G (p.Tyr261*), continued the pregnancy and delivered at term with a birth weight of 2850 g. Among the seven cases in which pathogenic or likely pathogenic variants associated with the FGR phenotype were detected by trio-WES, six opted for pregnancy termination. These included autosomal dominant disorders caused by mutations in the *WHSC1*, *FGFR3*, *TUBB2B,* and *BRPF1* genes (Cases 13, 14, 17, and 18), an autosomal recessive disorder due to compound heterozygous mutations in the *LIG4* gene (Case 15), and a case with maternal UPD(6) in combination with a pathogenic variant in the *FGFR2* gene (Case 20). Case 16, carrying the heterozygous variant c.84C>A (p.Cys28*) in the *IHH* gene and the heterozygous variant c.783C>G (p.Tyr261*) in the *ENPP1* gene, chose to continue the pregnancy and delivered at term with a birth weight of 2850 g. In another four cases in which pathogenic or likely pathogenic variants not directly linked to the FGR phenotype were detected, the pregnancies were continued. These involved variants in the *SMAD6*, *FLNB*, *MSH2*, and *BRIP1* genes, all resulting in term deliveries with birth weights ranging from 2330 g to 2700 g. All five live-born infants exhibited normal development during follow-up from 6 months to 2 years of age. In Case 20, the detection of a likely pathogenic variant in the *FGFR2* gene combined with maternal UPD(6) led the couple to decide on pregnancy termination. Case 19 opted for termination due to maternal hyperphenylalaninemia. Following termination, Case 19 was subjected to multidisciplinary management involving a dietitian, pharmacist, geneticist, and obstetrician to regulate phenylalanine levels and prepare for future pregnancies. As of the last follow-up, the patient had not conceived again.

## 4. Discussion

The genetic etiology of FGR is complex, involving chromosomal aneuploidies, CNVs, UPD, and single-gene mutations [39,40]. In this study, karyotype analysis detected three cases of chromosomal abnormalities (2.0%, 3/153), indicating that large chromosomal abnormalities remain a significant cause of FGR even in the absence of obvious structural anomalies. However, the aneuploidy detection rate in this study was lower than that reported previously (reported rates for large chromosomal abnormalities range from 3.9% to 9.4% [9,11,15]. This discrepancy is likely attributable to the routine implementation of non-invasive prenatal testing (NIPT) in the first trimester in this region. NIPT can effectively detect large chromosomal abnormalities at an earlier gestational stage, thereby reducing their frequency in FGR cases.

CNV is a significant genetic component underlying FGR. By altering gene dosage, CNVs can trigger gene dosage effects, thereby affecting fetal growth and development. In this study, all karyotypic abnormalities were detected by CMA. Furthermore, CMA increased the diagnostic yield by 5.9% by identifying CNVs and UPD that were missed by conventional karyotyping. These findings are consistent with data reported in the literature [12,13,41,42]. Previous studies have identified Xp22.31 microdeletions [13,42] and 17p12 microduplications [12] in isolated FGR cases. In agreement with prior reports, one case of Xp22.31 microdeletion and one case of 17p12 microduplication were also detected in the present study. UPD refers to the inheritance of both homologous chromosomes from a single parent, which can lead to abnormal expression of imprinted genes and subsequently impact fetal growth [43,44,45]. In this study, the SNP array detected one case of UPD(6) and one case of segmental UPD(16), consistent with previous literature [39,46,47]. Genetic counseling regarding pathogenic or likely pathogenic CNVs significantly influences couples’ decisions regarding their pregnancies. For instance, in Case 7, a maternally inherited 17p12 microduplication (associated with Charcot–Marie–Tooth disease type 1A) was identified, and in Case 12, a 15q11.2 microdeletion with reduced penetrance was found. After detailed genetic counseling of the clinical significance and penetrance of these chromosomal variants [40,48], both couples opted to continue their pregnancies.

This study provides a demonstration of the efficacy and sensitivity of trio-WES in isolated FGR cases, particularly for sequential application following a negative chromosomal microarray analysis (CMA). Trio-WES detected pathogenic variants in 14% of CMA-negative cases, including UPD, which is undetectable by CMA. For instance, in Case 20, trio-WES identified maternal UPD(6), which was missed by CMA. In this case, oligonucleotide-based CMA failed to detect UPD(6), while methylation-specific multiplex ligation-dependent probe amplification (MS-MLPA) revealed increased methylation signals in the 6q24.2 region with a normal copy number, suggesting maternal UPD(6). It is important to note that the SNP array cannot detect heterodisomic UPD (hetero UPD), further highlighting the limitations of CMA in UPD identification. This finding is consistent with previous research conclusions that CMA has inherent limitations in detecting UPD, and approximately one-third of molecularly confirmed UPD cases cannot be identified by CMA alone [44,49,50,51]. CMA based on single nucleotide polymorphisms (SNP) is primarily designed to detect copy number variations and regions of homozygosity; however, it has inherent limitations in identifying heterodisomic UPD—where both homologous chromosomes are inherited from a single parent but are not identical. In contrast, trio-WES, utilizing sequencing data from both parents and the fetus, enables direct allele phasing and detection of non-Mendelian inheritance patterns, including isodisomic and heterodisomic uniparental disomy. This methodological advantage allows trio-WES to identify UPD events that are missed by CMA. Trio-WES also detected seven pathogenic or likely pathogenic variants not directly associated with the FGR phenotype. Although these variants are not directly linked to FGR, they may have significant implications for the long-term health of the fetus. For instance, in Case 20, UPD(6) was considered the genetic etiology of FGR, while the incidentally identified heterozygous variant c.1032G>A (p.Ala344=) in the *FGFR2* gene in the same case directly influenced the couple’s pregnancy decision due to the presence of *FGFR2*-related disorders. The *MSH2* gene mutation detected in Case 23 is associated with Lynch syndrome, which may increase the risk of colorectal cancer in the fetus during adulthood. In Case 19, the fetal heterozygous *PAH*:c.1197A>T (p.Val399=) variant was not the cause of FGR. However, trio-WES revealed that the mother carried the pathogenic *PAH*:c.1197A>T variant in compound heterozygosity with c.1238G>C, indicating maternal hyperphenylalaninemia. This constitutes the genetic etiology for the current FGR and previous adverse pregnancy outcomes. As emphasized in the literature [52], this case highlights the importance of trio-WES in uncovering maternal genetic disorders that may affect fetal development. By identifying such conditions, trio-WES contributes to a more comprehensive understanding of the genetic components leading to FGR and can inform the development of individualized management strategies aimed at improving pregnancy outcomes. Therefore, trio-WES not only aids in the etiological diagnosis of FGR but also provides a more complete genetic information profile, thereby necessitating an expansion of the scope of genetic counseling. The prenatal diagnostic context amplifies the ethical weight of incidental findings. Unlike postnatal testing, the results are disclosed during a period of emotional vulnerability for the pregnant woman and under the pressure of decision-making regarding pregnancy continuation. This raises a series of critical issues concerning the scope of disclosure, the right not to know, and the management of parental anxiety. Our experience demonstrates that a multidisciplinary prenatal counseling team involving clinical geneticists, genetic counselors, maternal-fetal medicine specialists, and psychologists.

Our findings highlight that the combined application of trio-WES and CMA can effectively accelerate etiological diagnosis and guide clinical intervention. This sequential model—performing CMA first, followed by trio-WES for negative cases—enables the rapid detection of CNVs and aneuploidies while reserving whole-exome sequencing for in-depth investigation of monogenic disorders and epigenetic etiologies. This strategy significantly improves the diagnostic yield, which is crucial for prenatal decision-making and potential intervention.

Our data demonstrate that through subsequent trio-WES, a definitive genetic etiology for FGR was identified in seven fetuses (14% of the trio-WES subgroup), with an additional unexpected finding that untangled a maternal factor contributing to FGR. In total, an etiological diagnosis was established in eight cases (16%, 8/50). Although the per-test cost of trio-WES is higher than that of CMA, the adoption of a targeted sequential strategy—implemented only after negative results from CMA and karyotyping—can optimize resource allocation. This approach avoids the potentially substantial cumulative costs associated with undiagnosed cases, which may lead to long-term developmental support needs and familial recurrence risks.

## 5. Limitation

The small sample size (*n* = 153) and the trio-WES subgroup (*n* = 50) may reduce the statistical power for identifying rare genetic variants. All participants were recruited from a single tertiary medical center, which may introduce selection bias towards more severe phenotypes or specific demographic groups. The study was conducted exclusively in an Asian population, limiting the generalizability of the findings to other ethnic groups with different genetic backgrounds. The evaluated outcome measures were confined to pregnancy termination or neonatal status at birth; the long-term developmental, metabolic, and oncological risk profiles associated with incidental findings have not yet been elucidated.

Furthermore, this retrospective cohort did not systematically collect detailed maternal anthropometric data (e.g., pre-pregnancy body mass index, gestational weight gain). Future prospective studies should incorporate such maternal phenotypic information to better elucidate the interaction between the genetic component and constitutional factors in isolated FGR.

## 6. Conclusions

This study highlights the advantages of incorporating CMA and trio-WES into the prenatal diagnostic protocol for isolated FGR. Compared with traditional karyotyping, CMA demonstrates superior diagnostic capability, with a significantly higher detection rate for chromosomal abnormalities. Trio-WES enables the detection of monogenic disorders and enhances the identification of uniparental disomy (UPD), which may be missed by CMA. The combined application of these genetic technologies allows for a more comprehensive assessment of the genetic etiology of isolated FGR. Furthermore, trio-WES facilitates the identification of potential maternal genetic disorders that may affect fetal development. By detecting these conditions, trio-WES can inform the development of improved management strategies, thereby potentially improving outcomes in subsequent pregnancies.

However, the detection of incidental findings through trio-WES underscores the importance of multidisciplinary genetic counseling in addressing fetal growth issues and potential long-term health impacts, while also helping to alleviate anxiety associated with such incidental findings. Future research should prioritize cost-effectiveness analyses and the development of standardized guidelines for variant interpretation in the prenatal setting in order to further optimize clinical practice and improve patient outcomes.

## Figures and Tables

**Table 1:** Chromosomal abnormalities detected by karyotyping and CMA.

Case Number	GA # (Weeks)	Karyotype	CMA Results/Size	Outcome
1	20	mos 45,X[6]/46,XX[94]	arr[X] × 1~2	LB
2	18	47,XN + 21	arr[21] × 3	TOP
3	22	46,XY.del(11)(q24.2)	arr[GRCh37]11q24.2q25(127,699,535–137,937,416) × 1/10.2 Mb	IUFD

Abbreviations: GA, gestational age; LB, live born; TOP, termination of pregnancy; IUFD, intrauterine fetal demise. #: GA at the diagnosis of FGR.

**Table 2:** Chromosomal microarray analysis results.

Case Number	GA ^#^ (Weeks)	CMA Results	Type of Aberration/Size	Inheritance	Classification	Syndrome	Pregnancy Outcome
4	22	arr[GRCh37]16p13.3p12.2(94,808 − 22,768,821) × 2 hmz	UPD/22.7 Mb	De novo	P	Segmental UPD(16)	LB, 37w5d, BW 1660 g, normal development at 3-year-old follow-up
5	22	arr[GRCh37]14q12(32,297,119–33,263,470) × 1	del/966.4 Kb	De novo	LP	/	IUFD
6	30	arr[GRCh37]1q43q44(240,983,677–245,476,176) × 1	del/4.5 Mb	De novo	P	1q43-q44 microdeletion syndrome, AD intellectual developmental disorder 22	TOP
7	22	arr[GRCh37]17p12(14,099,565–15,428,902) × 3	dup/1.3 Mb	Maternal	P	Charcot–Marie–Tooth disease type 1A (CMT1A) (OMIM 118,220)	LB, 34w3d, BW 1830 g, hypospadias, preeclampsia
8	22	arr[GRCh37]Xp22.33p22.31(168,552–6,387,288) × 1	del/ 6.2 Mb	De novo	P	Leri–Weill dyschondrosteosis (OMIM 127,300)/ X-linked chondrodysplasia punctata (CDPX1) (OMIM 302,950)	TOP
9	26	upd(6)mat.arr[GRCh37] 6p22.3p21.1(156,975–43,855,790) × 2 htz, 6p21.1q21(43,855,791–107,691,648) × 2 hmz, 6q21q27(107,691,649–170,914,297) × 2 htz *	Maternal UPD	De novo	P	UPD(6)mat	TOP
10	29	arr[GRCh37]15q13.2q13.3(31,042,916–32,509,926) × 1	del/1.5 Mb	De novo	P	15q13.3 deletion syndrome	TOP
11	18	arr[GRCh37]19q13.11q13.12(33,044,716–37,930,875) × 1	del/4.9 Mb	De novo	P	/	TOP
12	24	arr[GRCh37]15q11.2(22,770,422–23,082,237) × 1	del/312 Kb	De novo	P	15q11.2 deletion syndrome (OMIM 615,656)	LB, 39w3d, BW 2540 g, normal development at 1-year-old follow-up

Abbreviations: GA, gestational age; UPD, uniparental disomy; P, pathogenic; LP, likely pathogenic ^#^: GA at the suspicion of FGR. * For Case 9, the initial SNP-array result of the amniotic fluid DNA was arr[GRCh37]6q11.1q21(61,972,917–107,691,648); 6p21.1p11.1(43,855,791–58,726,706) hmz. Further analysis by trio-CMA revealed UPD(6) is of maternal origin.

**Table 3:** Trio-WES results.

Case Number	GA #	Gene	Transcripts	Variant	Origin	Inheritance	ACMG Classification	Zygosity	OMIM Phenotypes	Outcome
13	30	*WHSC1* *	NM_001042424.3	c.3411_3412delTC (p.Arg1138Ilefs*11)	De novo	AD	P	heterozygous	Rauch–Steindl syndrome (OMIM 619,695)	TOP
						IC			Wolf–Hirschhorn syndrome (OMIM 194,190)	
14	22	*FGFR3* *	NM_000142.5	c.1620C>A(p.Asn540Lys)	De novo	AD	P	heterozygous	Achondroplasia (OMIM 100,800); Hypochondroplasia (OMIM 146,000)	TOP
15	27	*LIG4* *	NM_206937.2	c.1271_1275 delAAAGA (p.Lys424Argfs*20)	Mat.	AR	P	heterozygous	LIG4 syndrome (OMIM 606,593)	TOP
				c.833G>T(p.Arg278Leu)	Pat.		P	heterozygous		
16	27	*IHH* *	NM_006208.3	c.84C>A(p.Cys28*)	Pat.	AD	LP	heterozygous	Brachydactyly, type A1 (OMIM 112,500)	LB, 40w6d, BW 2850 g, normal development at 2-year-old follow-up
		*ENPP1* ^@^	NM_002181.4	c.783C>G(p.Tyr261*)	Mat	AD	P	heterozygous	Cole disease (OMIM 615,522); Diabetes mellitus, non-insulin-dependent, susceptibility to (OMIM 125,853)	
17	31	*TUBB2B* *	NM_178012.5	c.350T>C(p.Leu117Pro)	De novo	AD	P	heterozygous	Cortical dysplasia, complex, with other brain malformations 7 (OMIM 610,031)	TOP
18	22	*BRPF1* *	NM_001003694.2	c.1218C>A(p.Tyr406*)	De novo	AD	P	heterozygous	Intellectual developmental disorder with dysmorphic facies and ptosis (OMIM 617,333)	TOP
19	18	*PAH* ^@^	NM_000277.3	c.1197A>T(p.Val399=)	Mat.	AR	P	heterozygous	Phenylketonuria/Hyperphenylalaninemia, non-PKU mild (OMIM 261,600)	TOP
20	16	*FGFR2* ^@^	NM_000141.5	c.1032G>A(p.Ala344=)	De novo	AD	P	heterozygous	Antley–Bixler syndrome without genital anomalies or disordered steroidogenesis (OMIM 207,410); Apert syndrome (OMIM 101,200); Beare–Stevenson cutis gyrata syndrome (OMIM 123,790); Crouzon syndrome (OMIM 123,500); Jackson–Weiss syndrome (OMIM 123,150); LADD syndrome 1 (OMIM 149,730); Craniofacial–skeletal–dermatologic dysplasia (OMIM 101,600); Pfeiffer syndrome (OMIM 101,600); Saethre–Chotzen syndrome (OMIM 101,400); Bent bone dysplasia syndrome (OMIM 614,592); Scaphocephaly, maxillary retrusion, and impaired intellectual development (OMIM 609,579); Scaphocephaly and Axenfeld–Rieger anomaly; Craniosynostosis, nonspecific	TOP
		UPD(6) *^&^	/	Seq[GRCh37]6q23.3q27(400,000–171,000,000) × 2 hmz	Mat.		P	/	/	
21	30	*SMAD6* ^@^	NM_005585.5	c.1378dupG (p.Asp460Glyfs*105)	De novo	AD	LP	heterozygous	Craniosynostosis 7, susceptibility to (OMIM 617,439); Radioulnar synostosis, non-syndromic (OMIM 179,300); Aortic valve disease 2 (OMIM 614,823)	LB, 37w3d, BW 2550 g, normal development at 6-month follow-up
22	25	*FLNB* ^@^	NM_001457.4	c.6773-1G>A(p.Gln214*)	Pat.	AD	LP	heterozygous	Boomerang dysplasia (OMIM 112,310); Larsen syndrome (OMIM 150,250); Atelosteogenesis, type I (OMIM 108,720); Atelosteogenesis, type III (OMIM 108,721)	LB, 39w, BW 2630 g, normal development at 1-year-old follow-up
23	26	*MSH2* ^@^	NM_000251.2	EX1 Del	Pat.	AD	P	heterozygous	Lynch syndrome 1 (OMIM 120,435)	LB, 38w2d, BW 2330 g, normal development at 2-year-old follow-up
24	23	*BRIP1* ^@^	NM_032043.3	c.1565C>G(p.Ser522Ter)	Mat.	AD	LP	heterozygous	Breast cancer, early-onset, susceptibility to (OMIM 114,480)	LB, 39w2d, BW 2700 g, normal development at 2-year-old follow-up

Abbreviations: GA, gestational age; UPD, uniparental disomy; P, pathogenic; LP, likely pathogenic; mat, maternal; pat, paternal; AD, autosomal dominant; AR, autosomal recessive; IC, imprinting center. #: GA at the suspicion of FGR; *: variant related to the FGR phenotype; ^@^: incidental finding. ^&^: MS-MLPA showed an increased methylation ratio in the 6q24.2 region, with the copy number being normal, indicating UPD(6) mat.

## Data Availability

The original contributions presented in this study are included in the article. Further inquiries can be directed to the corresponding author.

## Abbreviations

The following abbreviations are used in this manuscript:

FGR	fetal growth restriction
CMA	chromosomal microarray analysis
WES	whole exome sequencing
CNVs	copy number variations
UPD	uniparental disomy
SNVs	single-nucleotide variants
P	pathogenic
LP	likely pathogenic
B	benign
LB	likely benign
VUS	variant of uncertain significance
GA	gestational age
LB	live born
TOP	termination of pregnancy
IUFD	intrauterine fetal demise
Mat.	maternal
Pat.	paternal
AD	autosomal dominant
AR	autosomal recessive
IC	imprinting center
MS-MLPA	methylation-specific multiplex ligation-dependent probe amplification
PCR	polymerase chain reaction
STR	short tandem repeat
